# The Origin of Defective Enamel

**Published:** 1884-10

**Authors:** W. H. Eames


AMERICAN JOURNAL

OF

DENTAL SCIENCE.

VOL. XVIII. THIRD SERIES—OCTOBER, 1884. No. 6.

ARTICLE I.

### THE ORIGIN OF DEFECTIVE ENAMEL.—Continued.

BY PROF. W. H. EAMES, D. D. S.

### DISCUSSION.

Opened by DR. L. C. INGERSOLL, of Keokuk, Iowa:

The subject presented by Professor Eames in his very able and excellent paper is too large to be entirely compassed in a discussion on an occasion like this. I can, therefore, but barely allude to several of his points, and pass on to the discussion of the main question, to account for the appearance of horizontal grooves and lines of pits on the labial, and sometimes on the lingual faces of the teeth also—including those cases where a succession of parallel grooves appear. Imperfect development of enamel, appearing in single spots, pits or wells in various locations on the teeth. I am willing to concede to congenital or pre-natal causes; for by the death or atrophy of a single cell, or a group of enamel cells, imperfect enamel would result. I shall not deny that suspended development may occur in the organs of tooth development, as in various other organs and tissues of the body—dwarfed limbs, missing toes, fingers, bones and teeth and other deformities are ample proof of this.

Dr. Eames is ingenious in his theory, and, in accordance with it, is able to account for some of the phenomena in question. But I do not see how he can account for the markings on first molars and bicuspids, nor for all the peculiarities of defect appearing in the enamel of the incisors. My object, however, is not so much to oppose him as to oppose the commonly accepted theory, and to establish another which, in my judgment, will sufficiently account for all observed cases. In this we agree, that the old theory is wrong; and I am glad to find so efficient a helper as Professor Eames in the overthrow of a theory so univeraally held by the profession, supported by the teachings of all our dental literature for more than a hundred years. Yet I am constrained to say that I have no idea that the repeated announcement of an acquiescence in the theory, as announced by Bourdet in 1757, is the result of repeated investigations and demonstrations of its truth. But when the theory of arrested development was once announced, the authority of the man, without demonstration, was sufficient to give it credence. So great was its plausibility that demonstration was not demanded, and in the absence of all other ætiological account concerning it, the theory of suspended development has met with universal acceptance, although to-day it remains, so far as I know, without a single valid argument to support it. The flimsiest evidence of its truth has been received as valid. Let us look at some of these so-called evidences. It is said that the teeth, hair, and nails, having the same histological origin, each alike, shows, at least in some cases, the effect of suspended development so apparent in the teeth, and, at the same time. A brief consideration will show the falsity of such assumptions. It is estimated that the life of nail cells is from four to five months, being less in summer than in winter. The nails being of perpetual growth, all markings of any sort on the nails would be obliterated in a period of a few months. How is it possible, then, that three years after the time when the teeth exhibit their markings similiar markings can be seen on the nails?

How is it, now, about the markings on the hair? Somebody in the long past, looking through his microscope, saw the imbricated condition of the surface, and at once pronounced it a case of suspended development, the same as appears on the teeth. If he had examined the hairs from a hundred heads, and from the bodies of the domestic animals, he would have found the evidence of measles or small-pox in every case. The structure of the human hair, as modern histological science gives it to us, is thus: [Blackboard illustration.] The hair of the cat is represented as a series of truncated cones, thus: [Blackboard illustration.] This imbricated condition, like the overlaping of shingles on a roof, is supposed to represent a period of suspended development.

About forty years ago there was published in New York a small work, giving an account of the formation and development of the teeth, and of various diseases to which they are subject. In this work the author introduces another illustration of suspended development, with results similar to those found on the teeth. The case was that of a cow's horn. He found it with alternating ridges and grooves, which he pronounced the result of faulty nutrition and arrested development. This went, so far as I know, as unquestioned an illustration as nails and hair. Suppose some doubter had ventured to ask the author, "How is it that these ridges and grooves never appear till after the animal is three years old?" The only wise answer he could have given and supported his theory would have been, that bovines never *suspend* till after they are three years old. Let us now, for a moment, look at the structure of a horn. Its corneous layers are also imbricated. Up to the third year the outer layer is continuous, from the tip to the skull. During the fourth year it runs out at the surface, and underneath its terminal edge is seen another layer. And thus, each succeeding year, is brought to view the terminal edge of one of the layers, and between these terminal edges is presented, from year to year, an additional groove.

The strongest and most reliable argument of all is of a historical character—"the mother of the child told me so." It is alleged that, in a large number of cases, in regard to which investigation has been made, the mothers and nurses have confirmed the theory of the investigator that, at the period when certain teeth were receiving their amelification, the child was afflicted with a severe constitutional disease.

You may fix any period during the first five years of the child's life—the years in which the teeth are in the process of amelification—and ask the mother, "Did not your child, at that time or during that year, have some constitutional disease?" and she will be compelled, in nearly every instance, to say "yes;" and that, too, not alone of the child whose teeth are pitted or otherwise marked, but of every child of her's. For see the long list of diseases to which children are subject at that period of life: Measles, whooping cough, eclampsia, scarlatina, eruptive fever, gastric fever, scalled head and a long list of exanthematous diseases. With this long catalogue of diseases on the tapis of childhood, it is scarcely possible for an enfeebled child to pass a single year and escape all. Hence, any mother could truthfully respond in the affirmative as to the happening of some constitutional disease during any assumed period that a professional man might name. I say *assumed* period; for with the known variation in the time of development of the teeth of different children, all is guess-work as to the particular stage of development of any child's teeth before they have emerged from the gum. Yet we are told by the wisest men of our land, and of all lands in the science of dentology, that these markings on the the teeth enable the dentist to tell, with considerable accuracy, the period when there was some constitutional disturbance. This is the same kind of accuracy with which Professor Tice predicted the weather. Turn over the pages of his almanac and you will find, at some specified date, "*Rain in places.*" So, if there is rain on that day any where on the face of the earth, he evokes all his thermal wisdom and astronomical science to declare that there must have been, at that time, "some *ærial* disturbance;" and when there is any ærial disturbance, there may be rain in places.

Now, how is it as to the testimony of mothers concerning the new doctrine, that these markings on the enamel occur *after* the teeth have cut the gum, and are brought about by chemical decomposition? *All* the diseases incident to childhood do not occur previous to five years of age. Several of the diseases named are quite as common an affliction between the ages of five and ten as between one and five. I have often asked mothers, when I have been consulted concerning this peculiar condition of the teeth of their children. Did not your child, at the time when these teeth were emerging, have some severe disease which involved the mucous membrane of the mouth? And I invariable get an affirmative answer. Thus far the testimony on both sides is equal, and therefore no proof of *either* theory.

Should I now get the testimony of observation and demonstration—which is not possible, or, if possible, has never been attempted concerning the old theory, but which is possible, and has actually been obtained concerning the new theory—according to the amount of this testimony the new theory is established. [A case related.]

Now let us scrutinize the accepted theory a little more closely. It is this, that at an early period in life, while the enamel is in the process of formation, a line of enamel cells, extending horizontally along the labial portion of the tooth, and, in some cases, completely encircling the crown, suspend the process of amelification, which is plainly marked on the fully emerged crown by a groove or by irregularly formed pits at intervals in the same line.

The deposit of enamel, as has been stated by Professor Eames in his former paper, is in one continuous sheet over the entire cap ot dentine. Why then should there be suspension only in a particular line of cells running horizontally? Why not just as often running vertically? Or why not a suspension of the developmental work of all the ameoloblasts of the enamel organ? Still further, why should we not expect to find the odontoblasts as well as the ameoloblasts affected in a similar manner, and thus find a groove or rather a hollow tube, as it must necessarily be, in the solid dentine entirely encircling the crown in some instances, opposite the groove on the enamel? Why are the markings sometimes on the labial faces and not on the lingual? Now, let us for a moment look at the most puzzling of all questions pertaining to the accepted theory, and that is to account for the appearance of *parallel* grooves at regular intervals, numbering, in some cases, five or six. The reasons sometimes offered are ludicrous, and if carried out to the full limit of their application they appear ridiculous in the extreme. One author, to whom I have alluded, introducing a goose quill for illustration—a quill having rings around the barrel of the quill, constricting it, at intervals as though threads had been tied around it and checked its filling out at these points to its full size—says that these rings, indicating suspended nutrition and develment, were probably caused by irregular feeding. At one time the farmer's corn gave out, and the goose almost starved; then again the fowl would have a full supply, and became sick and lose its appetite from overfeeding, which would result in another ring. Then, in accounting for the alternate rings and grooves on a cow's horn, he says they indicate the change of nutrition on the recurrence of summer and winter. In the Summer the animal is fat and sleek and well nourished, and the whole body develops symmetrically; but in winter the animal's food is changed—has no relish for the dry food, and perhaps is poorly sheltered and cared for, and this causes a suspended development in the horns, and probably in the hoofs too.

Now, what does the dentist say concerning the teeth? In accordance with the accepted theory he says: at this point of amelification the child had scarlatina; at that point whooping cough, when another groove was formed; at the next point small pox; at the next point eclampsia; and so on, through the list of constitution ailments of children that interrupt nutrition. And where it is observed that these grooves occur, in some cases, at regular distances from each others, all that he can say, in accounting for it, is that the child began with fits, and in exactly two months after he had an eruption fever; and in exactly two months after that he had small pox; and at regular intervals thereafter he had some other exanthematous disease, and wound up with a relapse of all he had gone through with before, which produced a suspension of development and a loss of enamel over a wide area. If there is anywhere to be found in ætiology anything more ridiculous than this, I should like to have it shown.

Now, if it is possible for you to disabused your minds of so erroneous a theory, after cherishing it long, you will be prepared to consider another theory and to mark its coincidence with well-known facts. The statement of the new theory is this, that these markings do not occur during the follicular development, but are the resuit of chemical action occuring after the development of the crown and after its emergence through the gum. The fact of the erosion of the enamel at the margin of the gums, in the form of a groove, is one of universal observation. It may be considered an accepted fact that a horizontal groove or line may thus be produced along the labial or buccal face of a tooth, and also, as sometimes seen, on the lingual faces. This dissolving of the enamel, in a horizontal line at the margin of the gum, may occur at any period duriug the emergence of the crown —at the time when the point of a cuspid has just made its appearance, or when half the crown is seen; in the latter case the marking will appear on the fully developed tooth, midway between the point of the cusp and the margin of the gum. The case is not now fully explained until we account for the appearance, at regular intervals, of grooves alternating with ridges.

I saw this difficulty some years ago, in attempting to establish the theory, and discovered, as I think, a full explanation in the physiological law that govern both plant and animal development, and that is the law of alternate vital action ; in other words, that *active* development, alternates with *arrested* development. Illustrations are both ample and evident throughout the body, and are apparent everywhere in animate nature. Look out now upon the maples, and you will see a case of suspended development ; the leaf buds of last year have fully unfolded ; the leaf has expanded to its full size ; you will see no more development at the top of the tree, possible, for two or three weeks. Is has suspended development at the ends of the branches, and is developing in some other part of the tree. After a period of arrested development at the top, you will see a renewal of development and the tree will be covered with a fresh growth of light colored, yellowish-green foliage. Look at the peach tree, to which I cannot point you this year, and when the petals of its blossom fall, the fruit germ will, in two or three days have gained the size of a pea, and soon after that of a cherry, and rapidly reach the size of a robin's egg, then suspend, and make no more increase in size for weeks. But this whole tree does not suspend ; it is developing in some other part, the stone which was before pulpy, is now hardening ; each part alternates with some other part.

Arrested development may, therefore, be shown to be a physiological law, and as such to prove the fallacy of assuming that arrested development implies constitutional disease and impairment of tissue. Because, under the physiological law, when the development processes are for a while suspended, the tissues do not suffer impairment by reason of the suspension. Nature takes up the work where she left off and the tissue is perfect throughout. Every part of the human body has its alternating part—the brain alternates in its development with the cranium ; the intestinal canal with the abdominal walls ; the teeth with the surrounding tissue, the alveolar walls ; the ameloblasts with the odontoblasts,—they are counterparts of each other.

The working out of this law is one of the economics of nature. The elements of bodily growth are obtained through the nutrient system. If it was required that all parts of the body should develop uniformly and at the same time, it would be impossibly for the nutrient system to supply all the elements. If all the hard tissue of the body developed equally, n the same period of time, it would be an utter impossibility, in the nature of things, to obtain in food the amount of lime salts which would be required. This fact shows the economy of nature in the law of alternate vital action. Were there never any functional disturbances caused by disease, the body would develop under this law in the same perfect symmetry as we might presume that it would under a system of uniform and uninterrupted development. This law of alternate development explains the incomplete formation of tissue, in cases of congenital hernia. In the alteration between the intestinal canal and the abdominal walls, the functions employed in the development of the latter may not keep pace with the intestinal development, caused by some functional weakness at the period when development should have been normally active in the abdominal tissue. We are often called to observe the want of harmony between the teeth and the jaws—the teeth are too large for the jaws, or the jaws are too large for the teeth. This occurs because, at the period when the vital forces were employed in the development of the one or the other of these tissue, either there was a deficiency in the supply of lime salts or a deficient in the power of assimilation.

Now, we are prepared to consider the alternation of grooves and ridges on the teeth. It must have been observed by some of you, at least, that investigators, concerning different organs of the body, have found it not only convenient, but necessary, so far as their observations concerning the teeth are concerned, if they would note decided changes and progress, to make their observations bi-monthly. This circumstance points to the fact that their alternating period of development returns about once in two months. This conforms, also, to the periods laid down in our books, as usually observed concerning the emergence of the teeth: a new tooth appearing about once in two months; the centrals of the deciduous set at about the seventh month; laterals at about the ninth month; then the molars appearing successively in the 24th, 26th, 28th and 30th months. This by-monthly development pertains not simply to the period of cutting, but also to the period of progress, on to the full development and normal position of the teeth in the jaws. Now, we are in possession of all the facts, individually considered, and necessary to the production of the peculiar pathological condition under consideration: *First fact*—an acrid fluid developed at the margins of the gum which, in its nascent state, is an active solvent of enamel; *Second Fact*—a *periodic* active development of the teeth, during which the tooth rapidly emerges, alternating with a periodic cessation of development, during which the tooth crown does not advance. The first fact may be the result of gastritis or a general febrile condition, whether associated with exanthematous disease or not. A diagnosis need not go beyond the inflammation of the mucous tissue of the mouth. Suppose, now, this occurs at the devel opmental period of from five to ten years of age, and is active at the alternating period of cessation of development, a horizontal groove must inevitable be the result. This period of cessation is followed, according to a law of nature, by a period of rapid development and emergence of the tooth crown from the gum. During this period there is no portion of the enamel sufficiently long in contact with the margin of the gum, where this erosive agent is working, to seriously effect the enamel. But if the diseased condition remains for several months through a summer season, as it sometimes does, when the next periodic cessation of development occurs, whether it is in exactly two months or three months, it matters not; at that period another groove will be formed, and the number of the grooves will correspond with the number of periods when there was a cessation of advance of the tooth towards the full exposure of the crown.

